# Emotions in the Brain Are Dynamic and Contextually Dependent: Using Music to Measure Affective Transitions

**DOI:** 10.1523/ENEURO.0184-24.2025

**Published:** 2025-07-03

**Authors:** Matthew E. Sachs, Mariusz S. Kozak, Kevin N. Ochsner, Christopher Baldassano

**Affiliations:** ^1^Center for Science and Society, Columbia University, New York, New York 10027; ^2^Departments of Music, Columbia University, New York, New York 10027; ^3^Psychology, Columbia University, New York, New York 10027

**Keywords:** emotion transitions, emotional dynamics, encoding models, fMRI, hidden Markov modeling; music

## Abstract

Our ability to shift from one emotion to the next allows us to adapt our behaviors to a constantly changing and often uncertain environment. Although previous studies have identified cortical and subcortical regions involved in affective responding, none have shown how these regions track and represent transitions between different emotional states, nor how such responses are modulated based on the recent emotional context. To study this, we commissioned new musical pieces designed to systematically move participants (*N* = 39, 20 males and 19 females) through different emotional states during fMRI and to manipulate the emotional context in which different participants heard a musical motif. Using a combination of data-driven (hidden Markov modeling) and hypothesis-driven methods, we confirmed that spatiotemporal patterns of activation along the temporoparietal axis reflect transitions between music-evoked emotions. We found that the spatial and temporal signatures of these neural response patterns, as well as self-reported emotion ratings, were sensitive to the emotional context in which the music was heard. In particular, brain-state transitions associated with emotional changes occurred earlier in time when the preceding affective state was of a similar valence to the current affective state. The findings argue that emotional changes are an essential signal by which the temporoparietal lobe segments our continuous experiences, and further clarify its role in linking changes in external auditory signals with our dynamic and contextually dependent emotional responses.

## Significance Statement

The emotions we experience in everyday life are rarely static; they fluctuate and transition in response to our ever-changing environment. However, little is known about the neural systems involved in the dynamic and contextually dependent nature of emotions. This paper addresses this issue by developing novel musical stimuli systematically designed to induce emotional reactions at specific timepoints. Using fMRI, we show that brain-state changes along the temporoparietal axis reflect transitions between music-evoked emotions. Furthermore, activation patterns associated with the same music were modulated by context, i.e., what was heard before. The findings argue that emotional changes are an essential signal for the brain when segmenting our continuous experiences and suggest a possible treatment target for cases of emotion dysregulation.

## Introduction

In everyday life, our emotions flexibly shift between states based on the temporal and social context (Trampe et al., 2015). Our ability to move from one emotional state to the next in response to our environment is crucial for our well-being, and our ability to understand and predict these emotion transitions in others is crucial for forming social connections (Thornton and Tamir, 2017). Despite this, neuroimaging studies that have addressed the dynamic quality of emotions have largely focused on identifying brain regions that track the rise and fall of a single emotional state in response to a stimulus (e.g., a film clip) or along a prescribed affective continuum (e.g., happy to sad; Goldin et al., 2008; Zaki et al., 2009, 2012). They cannot tell us if the cortical and subcortical regions that respond to isolated emotional content (Lindquist et al., 2012; Kragel and LaBar, 2015) also mediate transitions between qualitatively different emotions, nor how previous emotional states might influence the processing and representation of the current emotional stimulus (Manstead et al., 1983; Szpunar et al., 2012).

To bridge this gap, we partnered with musical composers to develop novel musical stimuli designed to induce a particular emotional reaction at specific periods of time (herein called events). Music can reliably express and elicit a range of emotions in the absence of language of visual information (Cespedes-Guevara and Eerola, 2018; Cowen, 2020), making it a particularly useful stimulus for assessing the generalizability and ecological validity of our current brain models of emotions (Bottenhorn et al., 2019) and their implications for typical and atypical nonverbal emotional understanding (Van Rheenen and Rossell, 2013). By working directly with composers, we were able to a priori define which musical instruments would be used, the emotions we wish to induce, the timing of the transition between those emotions, and the ordering. In this way, we could more effectively tease apart the various factors (context, low-level acoustic features, etc.) that drive emotional experiences and the associated neural responses.

Using these novel pieces of music, fMRI, and a combination of hypothesis-driven and data-driven statistical approaches, we addressed two main research questions. First, we asked which brain regions track emotional state transitions in response to music. For this, we compared voxel pattern stability within- versus across-emotional events (hypothesis-driven; Baldassano et al., 2018). We additionally used hidden Markov models (HMMs) to probabilistically identify brain-state transitions without using any timing information about the stimulus itself (data-driven; Vidaurre et al., 2017). Shifts in the stability of brain activation patterns within cortical brain structures (including the posterior medial cortex, tempoparietal junction, angular gyrus, inferior frontal cortex, and tempoparietal axis) have been previously shown to reflect both high- and low-level changes in narrative (Baldassano et al., 2017), musical structure (Sridharan et al., 2007; Farbood et al., 2015; Williams et al., 2022), and feelings of uncertainty in response to a movie (Majumdar et al., 2023). Here, we hypothesized that emotion transitions induced by our music would be a primary driver of these brain-state changes, as well as in regions known to be involved in emotion processing more generally, including the dorsomedial and ventromedial prefrontal cortex, insula, amygdala, putamen, pallidum, and caudate.

Second, we asked how emotional context influenced event representations and dynamics. When designing the musical stimuli, we created two versions of each piece, so that the same musical events could be heard in different contexts, i.e., preceded by a different emotion in each version. This allowed us to test how the brain pattern associated with a particular emotional event was influenced by the emotion of the preceding event. A previous study that used such an approach with movies found that semantic context and narrative framing can modulate the neural representations of the same stimulus in temporal and prefrontal cortices (Chen et al., 2017). We therefore predicted that the cortical regions identified above would show systematic alterations in spatial patterns based on what preceded the current event. Finally, using HMMs to detect the timing of neural event shifts (Lee et al., 2021; Cohen et al., 2022), we tested whether temporal patterns of brain-state transitions were impacted by the preceding emotion. Given that previous studies have shown that fluctuations in BOLD signal in subcortical (amygdala, hippocampus) and cortical (insular, temporal and prefrontal cortices) can persist to bias how new, unrelated information is encoded (Tambini et al., 2017; Tambini and Davachi, 2019; Clewett et al., 2020a), we hypothesized that both subcortical and cortical regions would demonstrate slower event transitions when the preceding event was of a contrasting valence.

## Materials and Methods

### Stimuli development

Three film composers wrote two original pieces of music. Each piece was divided into “events,” where each event conveyed a single emotional category that music has been shown to reliably induce (Cowen, 2020): sad/depressing, anxious/tense, calm/relaxing, joyous/happy, and dreamy/nostalgic. Each emotional category was expressed 6–7 times across the two pieces, using different musical elements during each recapitulation, but always the same four instruments (violin, piano, guitar, cello), for a total of 32 events. The length of each event was between 27 and 72 s. The tempo was set to be the same as (or a multiple of) the fMRI pulse sequence (80/160 BPMs). The 32 events were then divided into two distinct pieces (A and B), each with 16 unique musical events (4 emotions × 3–4 examples, ∼15 min in length). Musical interludes (4–12 s) were written by the composers to musically link one event to the next. To further increase the suitability of our music for MRI, we transposed each full piece a whole step down from where it was originally written, to match the fundamental frequency of the repeating currents of the SIEMENS MRI. Additional increases of the gain within certain bandwidths were adjusted as needed to allow for a more optimal listening experience with the headphones inside the MRI.

**Figure 1.**
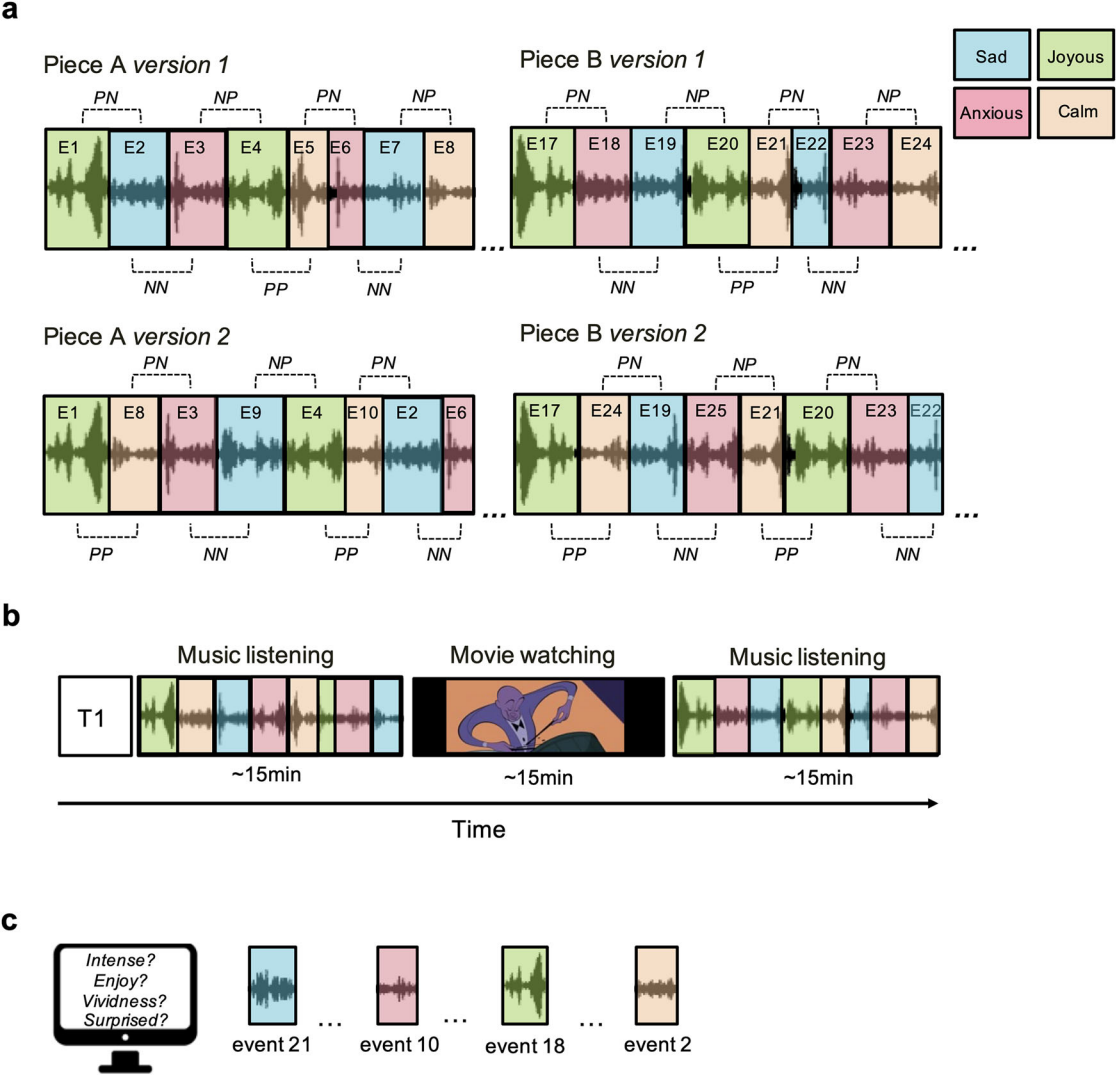
Stimuli and study design. ***a***, Schematic illustration of the two novel musical compositions. Each piece has 16 emotional events and two versions. Each version has the same events but in a different order so that any event that was previously preceded by a contrasting valence (e.g., NP) was now preceded by the same valence emotion (e.g., PP), and vice versa. ***b***, Design of the fMRI scanning session in which participants listened to one version of Piece A and B (version randomly selected and counterbalanced across participants). ***c***, Postscanning recall measures. P, positive valence; N, negative valence; PP, positive to positive valence emotion transition; NN, negative to negative valence emotion transition; PN, positive to negative valence emotion transition; NP, negative to positive valence emotion transition.

The ordering of events was constructed to ensure that the number of events preceded by an exemplar of the same valence (happy and calm considered positive and sad and anxious considered negative) was equal to the number of events preceded by an exemplar of a contrasting valence (Fig. 1*a*). Given that nostalgia is considered a mixed emotional state with both positive and negative aspects (Newman et al., 2019), we did not have specific hypotheses about its effect on subsequent emotional events and therefore only included four of the dreamy/nostalgia clips, one at the beginning and end of each piece. When excluding nostalgia, the final pieces had 14 emotion transitions of the same valence (positive to positive or negative to negative) and 14 emotion transitions of contrasting valence (positive to negative or negative to positive) across the two pieces (7 of each type in each piece). From this initial set of two pieces, the composers created an alternative version of each, using the same events, but reordered them so that any event that was previously preceded by a contrasting valence was now preceded by a same valence emotion, and vice versa (see Table 1 for the order and timing of each piece).

**Table 1.** Event order and timing for both versions of the two musical pieces

Event no.	Emotion	Emotion no.	Onset	Offset	Duration	Event no.	Emotion	Emotion no.	Onset	Offset	Duration
Piece A, Version 1						Piece A, Version 2					
1	Dreamy/nostalgic	1	0	60	60	1	Dreamy/nostalgic	1	0	60	60
2	Calm/relaxing	1	69	121	52	2	Joyous/cheerful	1	69	120	51
3	Joyous/cheerful	1	132	183	51	3	Calm/relaxing	1	129	181	52
4	Sad/depressing	1	192	240	48	4	Sad/depressing	1	194	230	36
5	Anxious/tense	1	249	297	48	5	Anxious/tense	1	234	291	57
6	Joyous/cheerful	2	306	360	54	6	Joyous/cheerful	2	300	348	48
7	Calm/relaxing	2	368	408	40	7	Calm/relaxing	2	357	393	36
8	Anxious/tense	2	417	474	57	8	Anxious/tense	2	402	441	39
9	Sad/depressing	2	485	521	36	9	Sad/depressing	2	447	495	48
10	Calm/relaxing	3	525	561	36	10	Calm/relaxing	3	506	546	40
11	Joyous/cheerful	3	570	618	48	11	Joyous/cheerful	3	555	609	54
12	Sad/depressing	3	627	675	48	12	Anxious/tense	3	615	663	48
13	Anxious/tense	3	684	723	39	13	Sad/depressing	3	672	720	48
14	Joyous/cheerful	4	730	768	38	14	Calm/relaxing	4	729	780	51
15	Calm/relaxing	4	774	825	51	15	Joyous/cheerful	4	789	827	38
16	Dreamy/nostalgic	2	834	894	60	16	Dreamy/nostalgic	2	834	894	60
Piece B, Version 1						Piece B, Version 2					
1	Dreamy/Nostalgic	3	0	36	36	1	Dreamy/nostalgic	3	0	36	36
2	Sad/depressing	4	45	93	48	2	Anxious/tense	4	45	93	48
3	Anxious/tense	4	102	150	48	3	Sad/depressing	4	102	150	48
4	Calm/relaxing	5	159	210	51	4	Joyous/cheerful	5	160	208	48
5	Joyous/cheerful	5	219	267	48	5	Calm/relaxing	5	219	270	51
6	Anxious/tense	5	279	327	48	6	Anxious/tense	5	279	327	48
7	Sad/depressing	5	336	390	54	7	Sad/depressing	5	336	384	48
8	Joyous/cheerful	6	399	426	27	8	Calm/relaxing	6	393	459	66
9	Calm/relaxing	6	435	501	66	9	Joyous/cheerful	6	468	495	27
10	Sad/depressing	6	510	558	48	10	Sad/depressing	6	504	558	54
11	Anxious/tense	6	567	615	48	11	Anxious/tense	6	567	615	48
12	Calm/relaxing	7	625	672	47	12	Joyous/cheerful	7	627	675	48
13	Joyous/cheerful	7	678	726	48	13	Calm/relaxing	7	684	728	44
14	Anxious/tense	7	735	765	30	14	Sad/depressing	7	735	807	72
15	Sad/depressing	7	771	843	72	15	Anxious/tense	7	816	846	30
16	Dreamy/nostalgic	4	852	894	42	16	Dreamy/nostalgic	4	852	894	42

Onset, offset, and duration are in seconds.

### Stimuli validation

To validate that the events induced the intended emotions at the intended time, subjective and continuous emotional responses from listeners were collected via a custom open-source web application (http://www.jonaskaplan.com/cinemotion/). The tool instructed participants to listen to a piece of music and to think about what emotion they felt in response (not how they think the performer/composer is feeling). When they felt a response, participants were instructed to select one of five possible buttons (happy, sad, anxious, nostalgic, and calm) to “turn on” that emotional label and to press it again to “turn off” that emotional label when they no longer felt that particular emotion. More than one label could be kept at a time. The label, onset time, and offset time were recorded continuously. Each participant listened to only one of the four possible pieces, divided into three ∼5 min sections with a self-paced break in between each to maintain focus. The breaks occurred within the middle of an emotional event, not at transition points. In addition, at the end of the final section, the ending of the piece transitioned into a completely different piece (Blue Monk by Thelonious Monk). This contrast was used as an attention check: any participant who did not press any button within 1.5–5.7 s after the transition to this new piece was removed from the analysis. Forty online participants were recruited via Prolific (https://www.prolific.co) to listen to each of the four pieces (160 total).

After removing participants who did not press any buttons for the duration of the piece or failed the attention check, we analyzed the ratings from 36 people who heard Piece A Version 1, 36 people who heard Piece A Version 2, 35 people who heard Piece B Version 1, and 35 people who heard Piece B Version 2. To determine if participants were significantly more likely to press the emotion buttons during the transition periods than not, we counted the number of raters who turned on or off any emotion label at the moments of transitions between emotional events, as identified by the composer. We then randomly shuffled the location of the transition period 1,000 times and, for each permutation, recalculated the number of people who pressed any button during those randomly shuffled transition times. The number of participants who had pressed any button during the real transition period was then compared with this null distribution of shuffled transitions to calculate a *z*-statistic and *p*-value.

To determine if the composer-defined emotion labels for each piece matched how the participants felt when listening, for each event, we counted the number of participants who had selected the intended emotion at any point during the duration of the event and averaged across-emotional categories. We then shuffled the emotion label of each event and recalculated the number of participants who had selected the emotion corresponding to this now randomly shuffled label 1,000 times. The number of people that had selected the composer-intended emotion, averaged across each emotion label, was then compared with this null distribution of shuffled emotion labels to calculate a *z*-statistic and *p*-value.

The emotional efficacy of the musical stimuli has been further validated in a separate study of episodic memory, in which we showed that fluctuations in emotional states induced by the music can bias the temporal encoding process in much the same way as other, more external context shifts (McClay et al., 2023).

### Acoustic features of the musical events

Acoustic features were extracted for each of the 32 musical event using the *librosa* Python package (McFee et al., 2015): mean and SD of root mean squares (RMS, dynamics), mean and SD of the log of the attack phase of the envelope of the signal (articulation), mean and SD of the per frame chroma centroid/chromogram center (pitch/melody), and mean and SD of harmonic change between consecutive frame (Hwang et al., 2013). To assess the degree to which emotional aspects of the music were correlated with lower-level acoustic features, Pearson’s correlations were run between each acoustic feature and the retrospective recall emotion ratings averaged across each subject for each musical event. The extracted acoustic features were regressed out of the shared response model (SRM) feature space data (using the residuals from a linear regression model) in order to assess if the brain regions reflected emotion state transitions.

### fMRI power analysis

To determine the number of participants to recruit, we conducted a power analysis by simulating an fMRI signal that corresponded to the proposed experimental design (with 32 events per musical piece) and simulating fMRI noise using previously collected fMRI data with similar scanning parameters. Using the *fmrisim* module (Ellis et al., 2020), part of the open-source Python software package *Brainiak* (Kumar et al., 2020), we first measured the noise properties (drift, autoregressive, physiological, task-related noise, and system noise) in a published dataset of 17 participants watching the first 50 min of the first episode of BBC's Sherlock (Chen et al., 2017). In order to determine the overall signal-to-noise ratio, we created artificial datasets by combining a ground-truth event structure (in which all the timepoints within each movie event were set equal to the average pattern for that event) with varying levels of simulated fMRI noise. We fit the HMM to each of these datasets and measured the degree to which the ground-truth event boundaries were successfully recovered and identified the signal-to-noise level at which the HMM performance matched the observed performance on the real movie-watching data.

We then generated simulated fMRI (using the noise properties and signal-to-noise ratio identified above) for up to 50 different pseudosubjects. For each tested sample size, we ran an HMM on group-averaged data using *k* = 32 events (to match the number of events in the music) and calculated a *z*-statistic that quantified the strength of the relationship between HMM-identified state transitions and musical transitions. For each tested sample size, the entire process was repeated 100 times to calculate power, where power was equal to the fraction of times in which the *p*-value of the effect was less than alpha. The alpha level was set to 0.005 to account for multiple comparisons across different brain regions. With 40 pseudosubjects, a significant effect at this alpha level was found in 96% of the simulations, allowing us to estimate that *N* = 40 would provide 96% power to detect event boundaries of similar strength to those in previous naturalistic datasets.

### Participants

This study was preregistered on the Open Science Framework (https://osf.io/a57wu/). Human subjects were recruited at a location which will be identified if the article is published. Forty-six right-handed participants completed the fMRI study (23 females, mean age = 27.18 years, SD = 4.19). Participants received monetary compensation ($20/hour) for their time. This study was conducted under an approved study protocol reviewed by the (IRB to be identified if the article is published). Informed consent was obtained from all participants. Three participants were removed due to technical issues, and one participant was excluded due to excessive movement (>20% of TRs for a session exceeded a framewise displacement of 0.3 mm; Power, 2017). We additionally removed four functional runs in which participants showed particularly idiosyncratic brain data, operationalized as having an average pairwise ISC <2 SDs below the mean ISC across all participants. All analyses are from these 39 participants (19 females).

### Experimental design

During scanning, participants listened to two full-length pieces of music (A and B) with no explicit instructions other than to listen attentively and restrict movement as much as possible (Fig. 1*b*). Which version of Piece A and B, as well as the order of presentation of the two stimuli, was counterbalanced across participants? In between the two music-listening sessions, participants watched a ∼12.5 min audiovisual movie (Rhapsody in Blue from Fantasia 2000), which was used for functional alignment (Chen et al., 2015).

After scanning, participants completed a Qualtrics survey that utilized retrospective behavioral sampling (Brandman et al., 2021). For each of the 32 emotional events, we extracted three unique 10 s excerpts taken from the beginning, middle, and end of the emotional event. Participants then listened to one of these three clips (exactly one from each event they heard during scanning, i.e., 32 events) selected randomly and presented in a random order. They were then asked to focus their memory on the first time they heard that particular moment during scanning, including no more than a few moments before and after it and to rate (1) how “vividly” they remember this moment in the piece on a seven-point Likert scale. If they did remember that particular moment (ratings > 1), they were subsequently asked to rate (2) how “surprising/unexpected” that moment in the music was the first time they heard it, (3) how “happy/joyous” did that moment make them feel, (4) how “sad” did that moment make them feel, (5) how “anxious/tense” did that moment make them feel, (6) how “calm/relaxed” did that moment make them feel, (7) how “dreamy/nostalgic” did that moment make them feel, and (8) how much did they “enjoy” this moment of the piece. Participants additionally listened to one clip from the piece they did not hear as an attention check, for a total of 17 clips (Fig. 1*c*).

### fMRI data acquisition and preprocessing

MRI images were acquired on a 3  T Siemens Prisma scanner using a 64-channel head coil. T2*-weighted echoplanar (EPI) volumes were collected with the following sequence parameters (TR = 1,500 ms; TE = 30 ms; flip angle (FA) = 90°; array = 64 × 64; 34 slices; effective voxel resolution = 2.5 × 2.5 × 2.5 mm; FOV = 192 mm). A high-resolution T1-weighted MPRAGE image was acquired for registration purposes (TR = 2,170 ms, TE = 4.33 ms, FA = 7°, array = 256 × 256, 160 slices, voxel resolution = 1 mm^3^, FOV = 256). Each of the two music-listening scans consisted of 607 images (6 s of silence before the music begins, 896 s/597 images of music listening, followed by 9 s of silence at the end). The movie-watching scan was acquired with identical sequence parameters to the EPI scans described above, except that the scans consisted of 496 images (744 s).

MRI data were converted to Brain Imaging Data Structure (BIDS) format using in-house scripts and verified using the BIDS validator: http://bids-standard.github.io/bids-validator/. The quality of each participant's MRI data was assessed using an automated quality control tool (MRIQC v0.10; Esteban et al., 2017). Functional data were preprocessed using fMRIPrep version 20.2.1 (Esteban et al., 2019; https://zenodo.org/records/10790684), a tool based on Nipype (Gorgolewski et al., 2011; https://zenodo.org/records/581704, RRID:SCR_002502). Each T1w (T1-weighted) volume was corrected for intensity nonuniformity using N4BiasFieldCorrection v2.1.0 and skull-stripped using antsBrainExtraction.sh v2.1.0 (using the OASIS template). Brain surfaces were reconstructed using recon-all from FreeSurfer v6.0.1 (Dale et al., 1999; RRID:SCR_001847), and the brain mask estimated previously was refined with a custom variation of the method to reconcile ANTs-derived and FreeSurfer-derived segmentations of the cortical gray matter (GM) of Mindboggle (Klein et al., 2017; RRID:SCR_002438). Spatial normalization to the International Consortium for Brain Mapping 152 Nonlinear Asymmetrical template version 2009c (Fonov et al., 2009; RRID:SCR_008796) was performed through nonlinear registration with the antsRegistration tool of ANTs v2.1.0 (Avants et al., 2008; RRID:SCR_004757), using brain-extracted versions of both T1w volume and template. Brain tissue segmentation of cerebrospinal fluid (CSF), white matter (WM), and GM was performed on the brain-extracted T1w using FAST (FSL v5.0.9, RRID:SCR_002823; Zhang et al., 2001).

Functional data were motion corrected using MCFLIRT (FSL v5.0.9; Jenkinson et al., 2002). Distortion correction was performed using an implementation of the TOPUP technique (Andersson et al., 2003) using 3dQwarp (AFNI v16.2.07; Cox, 1996). This was followed by coregistration to the corresponding T1w using boundary-based registration (Greve and Fischl, 2009) with six degrees of freedom, using bbregister (FreeSurfer v6.0.1). Motion correcting transformations, field distortion correcting warp, BOLD-to-T1w transformation, and T1w-to-template (MNI) warp were concatenated and applied in a single step using antsApplyTransforms (ANTs v2.1.0) using Lanczos interpolation.

Physiological noise regressors were extracted by applying CompCor (Behzadi et al., 2007). Principal components were estimated for the two CompCor variants: temporal (tCompCor) and anatomical (aCompCor). A mask to exclude signal with cortical origin was obtained by eroding the brain mask, ensuring it only contained subcortical structures. Six tCompCor components were then calculated including only the top 5% variable voxels within that subcortical mask. For aCompCor, six components were calculated within the intersection of the subcortical mask and the union of CSF and WM masks calculated in T1w space, after their projection to the native space of each functional run. Framewise displacement (Power et al., 2014) was calculated for each functional run using the implementation of Nipype. Many internal operations of FMRIPREP use Nilearn (Abraham et al., 2014; RRID:SCR_001362), principally within the BOLD-processing workflow. For more details of the pipeline, see https://fmriprep.readthedocs.io/en/20.2.0/workflows.html.

Using the above output, noise components were regressed out of the data, including six scan-to-scan motion parameters (*x*, *y*, *z* dimensions as well as roll, pitch, and yaw), their derivatives, CSF and WM signal, framewise displacement, and the first five noise components estimated by aCompCor. High-pass temporal filtering (0.008 Hz) was applied using discrete cosine bases. The resulting whole-brain time series were then *z*-scored within subjects to zero mean and unit variance.

### ROI and searchlight definition

We used a multivoxel searchlight approach, in which data from circular groups of vertices on the cortical surface (radius 11 vertices/∼15 mm radius, with each vertex covered by 14 different searchlights) were iteratively selected. We additionally ran the respective analyses on 11 subcortical ROIs, including the left and right thalamus, striatum (caudate and putamen), pallidum, hippocampus, amygdala, and bilateral nucleus accumbens, as defined by the FreeSurfer subcortical parcellation.

To account for the fact that anatomical alignment techniques may be insufficient for aligning fine-grained spatial patterns across individuals, we used a shared response model (SRM) to functionally align data from each searchlight/ROI into a common, low-dimensional feature space (Chen et al., 2015). To avoid any potential issues around double-dipping, we fit the model using brain activation collected during a completely separate task, in response to an audiovisual movie without lyrics (Rhapsody in Blue from the movie Fantasia 2000) and then applied the fitted model to brain patterns recorded during the music-listening task. The model determines a linear mapping (from voxels to shared features) between an individual's functional response and a shared response that is well-aligned across subjects (Baldassano et al., 2018). Audiovisual movies have been shown to be especially effective in mapping out a maximal set of shared features, learning a mapping that generalizes well even to more restricted stimuli like the audio clips used in this experiment (Haxby et al., 2011). Given time by voxel data matrices *D_i_* from every subject, SRM finds a voxel × feature transformation matrix *T_i_* for every subject such that *D_i_* × *T_i_* ≈ *S*, where *S* is the feature time courses shared across all subjects. The transformations are chosen to maximize the similarity between corresponding timepoints. The number of features was set to be consistent across all ROIs/searchlights, independent of the number of voxels within the ROIs (10% of the size of the largest ROI, yielding 80 features).

### Statistical analysis

For all analyses below, a cluster threshold approach was performed to correct for multiple comparisons across searchlights (Stelzer et al., 2013). Specifically, we reran the particular analysis 1,000 times with shuffled data, calculated the number of adjacent vertices that were statistically significant at the *p* = 0.05 uncorrected cutoff (to form clusters), and took the max cluster size for each permutation. We then determined the cluster sizes of our real data in the same way and determined how many of those were >95% of the null clusters (cluster threshold = 0.05).

#### Brain regions that track transitions from one emotional state to another: hypothesis-driven approach

**Figure 2.**
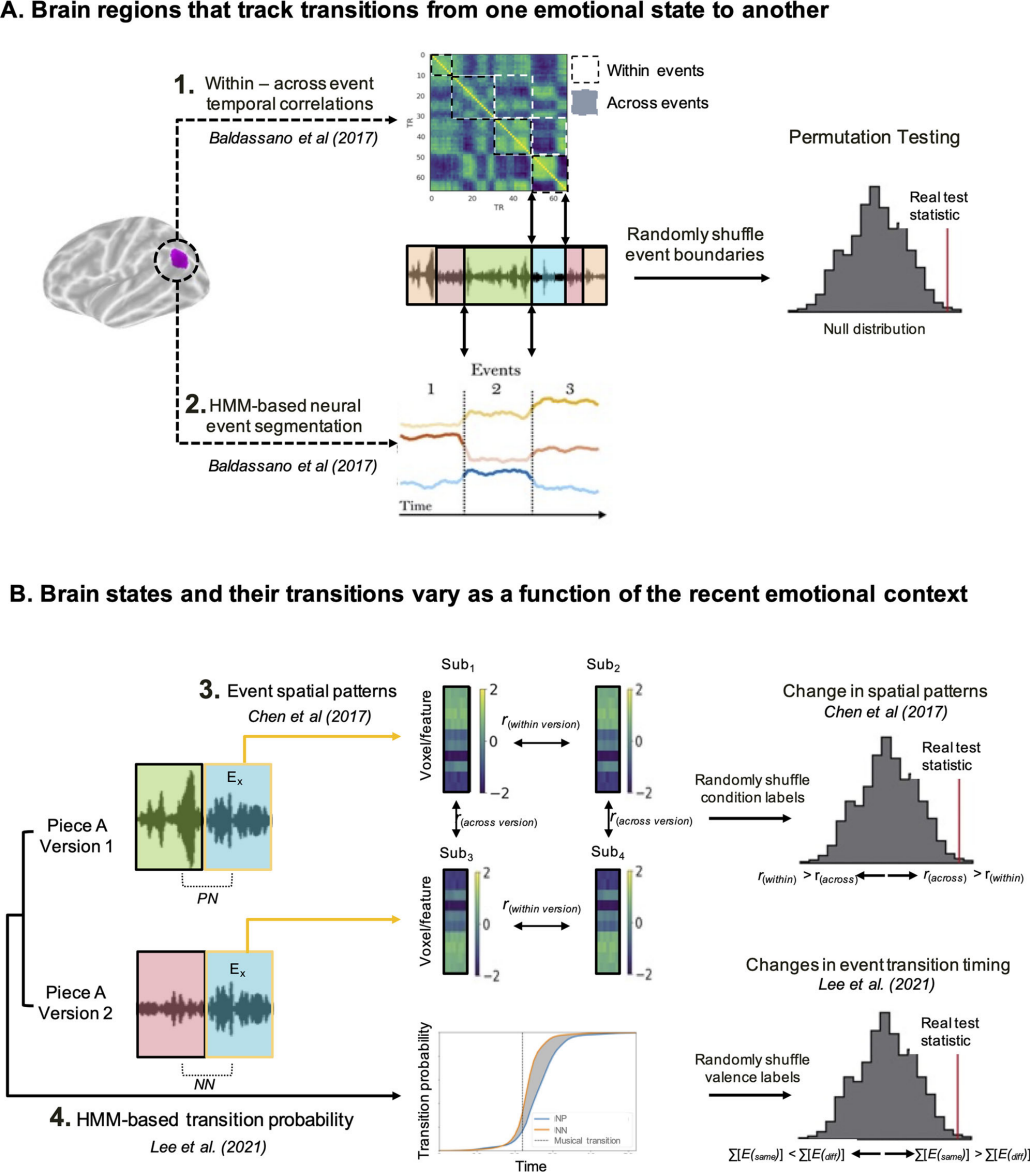
Flowchart of methodological approach. To answer the question of which brain regions track emotional transitions, for each searchlight or ROI in the brain, we used (***A1***), within-event versus across-event temporal correlations and (***A2***) HMM-based event segmentation. To answer the question of how these brain states vary by emotional context, we used (***B3***) pattern similarity analysis to identify regions that show systematic differences in spatial patterns based on condition (heard in the same version vs across versions) and (***B4***) HMM-derived probability distributions to identify brain regions in which the timing of a brain-state transition varies as a function of the preceding emotion.

If a brain region is sensitive to emotional transitions, then we would expect that the brain patterns at timepoints within a particular emotional event should look more similar than brain patterns at timepoints that cross emotional event boundaries (Baldassano et al., 2017). To this end, for each searchlight/ROI, the correlation between the patterns of shared features (from hyperalignment) was computed for all pairs of timepoints. We took the average correlation between all TRs that were within an emotional event and all TRs that spanned an emotional event (i.e., between event *X* and an adjacent event *X* + 1). The statistical significance of across- versus within-boundary correlations was determined by randomly shuffling the boundaries of events (preserving event lengths) and recalculating the correlations (Fig. 2*A*).

#### Brain regions that track transitions from one emotional state to another: data-driven approach

We supplemented the above findings with data-driven, generative models that try to learn latent states as well as their transitions based on the patterns of recorded brain activation (Baldassano et al., 2017). For this, we used the event segmentation model in the *Brainiak* Python package (Kumar et al., 2020). The package uses a variation of an HMM that assumes that participants experience a sequence of discrete events while processing a naturalistic stimulus, and each of these events has a discrete neural signature that is relatively stable, marked by a period of instability at transition timepoints. In this model, all states should be visited at least once, and all participants start at event *s*_1_ = 1 and end in a particular state *s_T_* = *K*, where *T* is the total number of timepoints and *K* is the total number of events. At each timepoint, the model can either advance to the next state or remain in the same one, which results in a transition matrix where all elements are zero, except for the diagonal and the adjacent off-diagonal. The diagonal probability (probability of remaining in the same state) is set to (*K*−1)/*T*, and this value is not adjusted when fitting the model. Importantly, the model does not assume that HMM-based event segmentation model events have the same length.

For each searchlight/ROI, the event segmentation model was applied to data in SRM space, averaged across all participants with the number of events set to the number of emotional transitions defined by the composers (16 for each piece). After fitting the HMM, we obtain an event-by-timepoint (16 × 597) matrix for each piece, giving the probability that each timepoint belongs to each event. For each TR, we took the entropy (using the scipy stats function) across the probability distribution, which tells us, for each TR, the likelihood of a boundary switch. We then calculated this entropy value at the moments of composer-defined transitions and determined if these values were greater than what would have been expected by chance through permutation testing. A null distribution of entropy values was created by shuffling the timing of the behavioral events (preserving their lengths) 1,000 times and recalculating. The average entropy at real event transitions was compared with null entropy values to calculate a *z*-statistic and a subsequent *p*-value.

#### Systematic changes in subjective reporting of feelings based on emotional context

To evaluate if the emotions we feel in response to a particular piece of music vary based on what we heard before, we first tested if self-reported emotion responses to the musical clips, recorded in the postscanning retrospective sampling, significantly varied as a function of condition (Version 1 vs 2). For each music clip, we calculated the correlations between ratings from pairs of participants who heard that clip within the same condition (both Version 1) as well as across conditions (one heard it in Version 1 and another in Version 2) and calculated differences in the mean of the within-condition pairwise correlations and the across-condition pairwise correlations.

In addition to the overall ratings, we tested if the context manipulation influenced the time it took for participants to report feeling the intended emotion of each clip. For this behavioral analysis, we used the data from the online, stimulus validation study. For each emotional event, we determined the timepoint when the number of participants who had “turned on” the intended emotion (pressed the corresponding button) reached 90% of the maximum number who turned on that emotion at any point. Excluded the first even of each piece, we then categorized all other events across the two pieces into one of four conditions: a positive emotional event preceded by a negative emotional event (NP), positive emotion preceded by a positive emotion (PP), negative emotion preceded by a positive emotion (PN), and negative emotion preceded by a negative emotion (NN). To determine significant differences in this timing across conditions (e.g., PP vs NP), we created a null distribution by randomly permuting whether or not the event was preceded by a negative or positive emotion and comparing the actual differences in time to peak to the random distribution of differences between conditions.

#### Systematic changes in spatial patterns of brain activation for emotional events based on context

If the emotional context systematically alters affective brain representations, then pairs of subjects who experienced an event with the same preceding event (both heard it in Piece A Version 1, for instance) should show have more similar patterns of activation than pairs of participants who heard the same event with different preceding events (one heard it in Piece A Version 1 and the other in Piece A Version 2; Chen et al., 2017). To test this, within a particular ROI/searchlight, we averaged the data across timepoints within each event, resulting in one pattern of SRM feature-wise activity per each emotional event. We then split participants into groups depending on which version of the two pieces they heard, that is, if the context in which that participant heard an emotional event was preceded by the same valence or a contrasting valence, and calculated split-half correlations between all combinations of group-averaged data (same context: *r*_(A1H1,A1H2)_, *r*_(B1H1,B1H2)_, *r*_(A2H1,A2H2)_, *r*_(B2H1,B2H2)_; different contexts: *r*_(A1H1,A2H1)_, *r*_(A1H2,A2H2)_, *r*_(A1H1,A2H2)_, *r*_(A1H2,A2H1)_, and four more for version B). We then calculated the average (geometric mean) of the correlations across all events within each grouping and tested if the average spatial correlations for emotional events that were heard in the same context (within the same version) were significantly greater than those heard in different contexts [across versions; mean(geometric_mean(*r*_(A1,A1)_ *r*_(A2,A2)_), geometric_mean(*r*_(B1,B1)_ *r*_(B2,B2)_)) > mean(*r*_(A1,A2)_, *r*_(B1,B2)_)].

To determine significance, we performed a permutation analysis wherein the participant condition labels (whether each participant heard Version 1 or Version 2 of the pieces) were randomly shuffled 1,000 times at the full piece level. Then, for each condition within each shuffling, cross-subject pairwise correlations were recalculated, and the means of cross-subject correlations binned according to the same groupings as above (now randomized) were recalculated. The true difference in correlation values was compared with the random null distribution to determine significance, and resulting statistical maps were cluster-thresholded.

To avoid the possibility that differences were an artifact of fMRI signal spilling over from the event before (arbitrarily resulting in pairs of participants who heard the piece in the same version appearing to have activation patterns more similar due to the signal coming from the same previous event), the analysis was run using only data averaged across the second half of each event, that is, not including any data that were temporally close to an emotion boundary/transition.

#### Systematic shifts in timing of transitions based on context

If certain emotional states can linger and influence subsequent stimuli processing (Tambini et al., 2017), then the time it takes for the brain to transition into a new emotional state should depend on what came before it. In order to measure the speed of the neural transition from one emotion to the next, we fit an HMM to group average data for both versions of each event, corresponding to the timepoints of that event as well as the timepoints of the musical event proceeding it. Since the length of the proceeding event could vary between versions of the piece, we cropped the longer of the two proceeding events to ensure that the number of timepoints inputted into each model was identical across conditions. For each model, the number of events (*k*) was set to 2.

At each timepoint, the HMM produces a probability distribution that describes the degree to which the model thinks the activity pattern at that timepoint reflects the current event or the previous event. Computing the expected value of this distribution therefore provides an index at each timepoint of the likelihood that the brain region pattern is reflective of the previous versus current state. The sum of the expectation values describes the total time that a particular brain region spends in the current versus previous event, with a greater sum indicating that the brain-state transitioned sooner in time. If the sum of these expectation values is greater when a musical event is proceeded by one particular emotion versus another, this indicates that the preceded emotion influences the timing of the transition to the current emotional state (Lee et al., 2021).

We calculated the expected value of the event assignment at each timepoint (dot product of the probability function with event labels) and summed this value across timepoints (higher sums correspond to faster transitions; Fig. 2*B*). We categorized all summed values into one of two groups: same valence events (e.g., happy event preceded by a calm event, PP) or different valence events (e.g., happy event preceded by a sad event, NP). We then took the difference in this sum between the same valence (PP and NN) and different valence (NP and PN), which indicates the number of timepoints by which the event transition is shifted, which was subsequently converted into seconds by multiplying by the TR (1.5 s). To determine significance, a one-sample *t*-statistic (mean divided by standard deviation) was calculated from the distribution of same–different valence differences in sums. This value was compared with a null distribution created by randomly shuffling the valence labels and recalculating the difference between same and different conditions 1,000 times. This was done for each searchlight/ROI.

### Code and data accessibility

Upon acceptance of the manuscript, fMRI images in BIDS format will be published on OpenNeuro. We will also publish the musical stimuli and behavioral ratings as a dataset to be used by researchers interested in music and emotions. The code/software described in the paper is freely available online at (URL redacted for double-blind review). Audio files of the stimuli are made available on OSF (URL redacted for double-blind review).

## Results

### Assessing the validity of musical emotions and their transitions with independent ratings

Across all four pieces, the number of raters who turned ON/OFF an emotion at the transition points was significantly greater than at random timepoints (Piece A1 *z*-stat: 3.6, *p* = 0.0002; Piece A2 *z*-stat: 3.21, *p* = 0.0006; Piece B1 *z*-stat: 4.32, *p* < 0.0001; Piece B2 *z*-stat: 4.15, *p* < 0.0001). Furthermore, the average number of participants who had selected the composer-intended emotion label during all the timepoints within each emotional event was significantly greater than chance for all emotional categories, except for “nostalgia” (calm: mean = 17.63, *z*-stat = 2.28, *p* = 0.01; happy: mean = 17.22, *z*-stat = 4.40, *p* < 0.001; sad: mean = 16.79, *z*-stat = 3.38, *p* < 0.001; anxious: mean = 19.16, *z*-stat = 5.53, *p* < 0.01; nostalgic: mean = 11.16, *z*-stat = 1.09, *p* = 0.14).

### Brain regions along the tempoparietal axis track transitions from one emotional state to another

**Figure 3.**
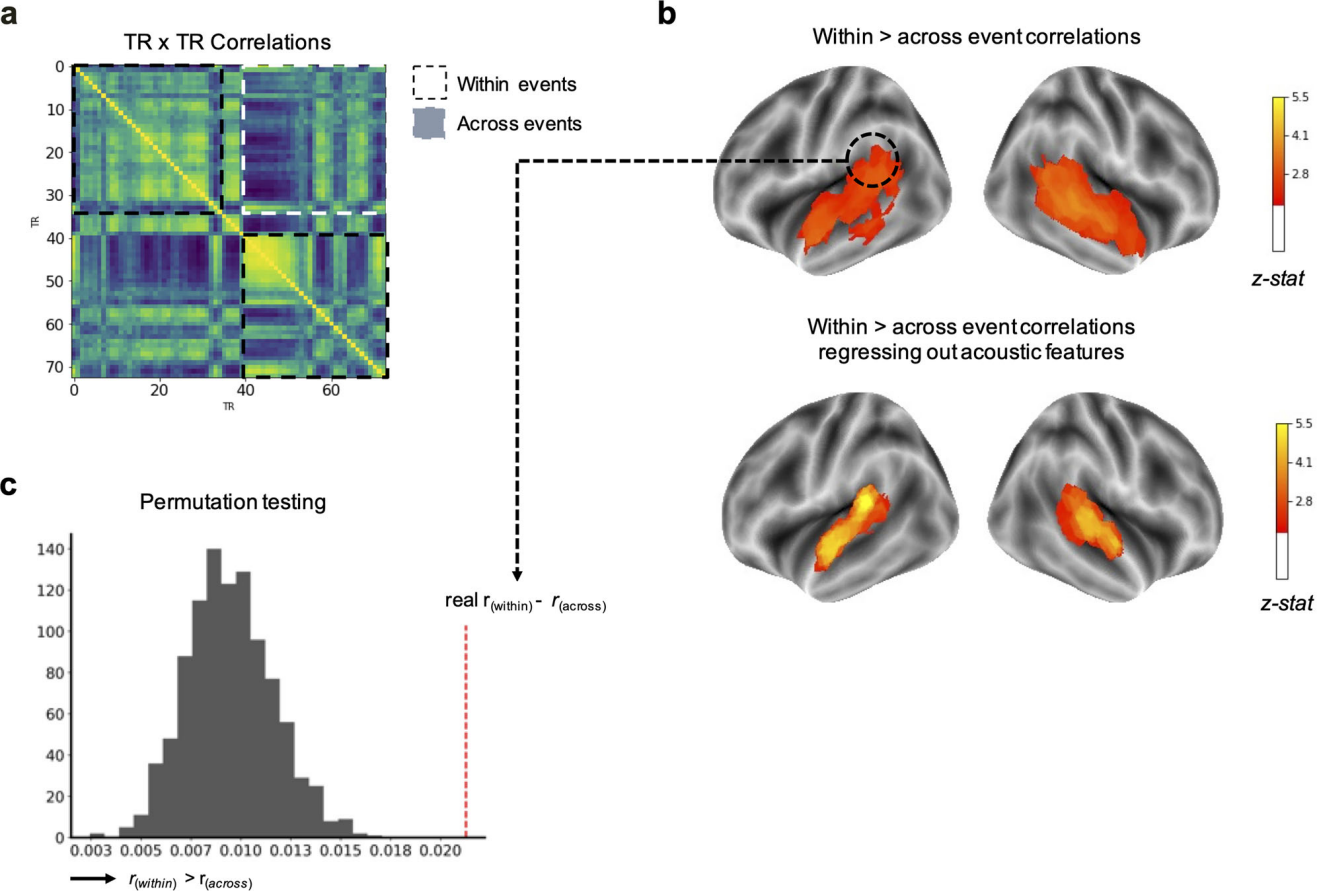
Brain-state changes driven by emotion transitions. ***a***, Truncated timepoint-by-timepoint correlation matrix (70 × 70), averaged across all participants, for the circled searchlight, demonstrating stronger within-event correlations compared with across-adjacent event correlations. ***b***, Brain regions in which timepoint-by-timepoint correlations were significantly greater within a composer-defined emotional event as compared with across-emotional events, even after acoustic features are regressed out. Colors correspond to *z*-scores across the cortical surface, relative to a (***c***) null distribution. Searchlights were cluster-corrected *p* < 0.05.

The difference between the within-event correlation and the across-adjacent event correlation indicates the extent to which a brain region's activation pattern shifted at event transitions. Regions that showed significantly higher correlations between timepoints within- versus across-emotional boundaries included the bilateral auditory cortex, superior temporal and middle temporal gyrus, and temporal pole as well as the left supramarginal gyrus and angular gyrus (Fig. 3*b*). We obtained confirmatory results using HMMs to detect how often the most salient neural pattern shifts aligned with composer-defined transitions, finding significant alignment in the bilateral superior temporal and middle temporal gyrus, left supramarginal gyrus, and angular gyrus.

#### Similar patterns are found when regressing out acoustic features

Because there is an expected, yet modest, correlation between subjective emotion ratings and acoustic features of the music (Table 2), the two analyses presented above were repeated on data with acoustic features regressed out of the SRM feature space data first using the residuals from a linear regression model (Yang and Chen, 2011; Williams et al., 2022). These features included timepoint-by-timepoint information related to dynamics (RMS), articulation (attack log), timbre (chroma centroid), and harmony (harmonic change). The hypothesis-driven largely mirrored the original findings and varied only in that the extent of the significant results in the temporoparietal cortex (Fig. 3*b*). Specifically, the right temporal pole and bilateral middle temporal gyrus no longer showed greater within-event versus across-event temporal correlations. Furthermore, matches between HMM-defined brain-state transitions and composer-defined emotion transitions were no longer significant in any part of the right hemisphere axis after acoustic features were regressed out of brain signal and were no longer significant in the left temporal pole and middle temporal gyrus.

**Table 2.** Correlation between subjective emotion ratings and key acoustic features extracted from each musical event

Acoustic feature		Calm	Happy	Sad	Anxious	Nostalgia
Volume	RMS	−0.388	0.247	−0.281	0.302	−0.033
Harmony	Tonnetz max	0.136	−0.124	0.203	−0.107	0.033
	Tonnetz (SD)	0.331	−0.070	0.273	−0.295	0.229
Timbre	Chroma (mean)	−0.285	0.088	−0.286	0.285	−0.168
	Chroma (SD)	0.208	−0.098	0.192	−0.120	0.097
	Chroma max	0.108	−0.079	0.101	−0.017	0.014
	Spectral centroid	−0.244	0.085	−0.110	0.174	0.016
	Spectral spread	−0.063	0.002	0.000	0.061	0.091
	Spectral roll-off	−0.226	0.092	−0.099	0.160	0.038
	Spectral novelty	−0.539	0.232	−0.392	0.411	−0.214

### Brain event pattern and their transition timings vary as a function of the recent emotional context

#### Emotional context influences how and when we feel

Using the postscanning retrospective ratings of emotional responses to each event, we found that within-piece pairwise correlation was significantly greater than the across-pieces pairwise correlations (*r*_within_ = 0.303, *r*_across_ = 0.265, *z*-stat = 2.0, *p* = 0.04), suggesting that subjective multivariate emotion ratings were systematically influenced by the prior emotional state. When averaging across-emotional label, the calm and sad clips varied the most by context (within *r*_happy_ = 0.37, across *r*_happy_ = 0.35; within *r*_sad_ = 0.20, across *r*_sad_ = 0.15; within *r*_calm_ = 0.19, across *r*_calm_ = 0.15; within *r*_anxious_ = 0.50, across *r*_anxious_ = 0.48).

In addition, when positive emotional events were preceded by the same valence, participants were on average 9 s faster to “turn on” that emotional state than when the same positive event was preceded by a negative event (difference valence), which was determined to be statistically significant (*z*-stat = 1.99, *p* = 0.04) based on a null distribution created by randomly permuting the valence of the previous emotion. This time to peak was 2.3 s faster for negative emotional events proceeded by same (negative) valence versus different valence (positive) events, though this difference was not statistically significant (*z*-stat = 0.49, *p* = 0.31) when compared with the null distribution.

#### Patterns in the auditory cortex systematically vary as a function of emotional context

**Figure 4.**
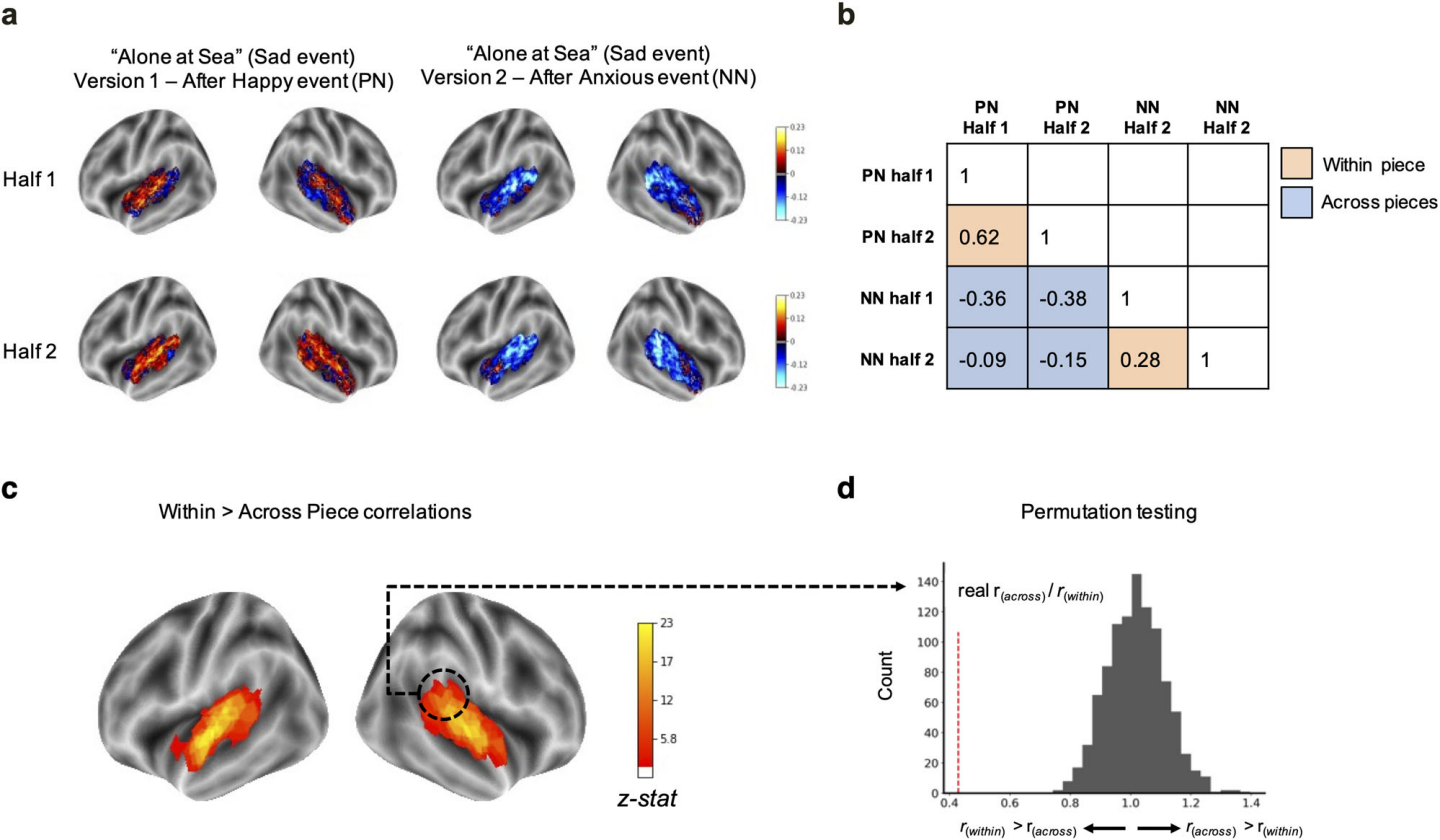
Context-based changes in spatial brain patterns. ***a***, Spatial patterns in the temporal lobe during the same musical event are more similar between users who heard that event in the same context/version as compared with across versions (red, positive normalized fMRI signal; blue, negative normalized fMRI signal). ***b***, Within- and across-condition split-half spatial correlations. ***c***, Brain regions in which spatial patterns were significantly more similar in pairs of participants who heard the emotional event in the same condition (all in the same version, i.e., within piece) as compared with pairs of participants who heard the music in different conditions (one in Version 1 and the other in Version 2, i.e., across piece). Colors correspond to *z*-stats of the ratio of across-group versus within-group correlations as compared with a (***d***) null model in which group membership was randomly permuted. Resulting statistical maps were cluster-corrected at *p* < 0.05.

Pairs of subjects who experienced an event within the same piece (e.g., both heard it in Piece A Version 1) had more similar patterns of activation than pairs of subjects who experienced the same event across versions (e.g., one heard it Piece A Version 1 and the other in Piece A Version 2; Fig. 4*a,b*). The brain regions that showed significantly greater within-piece pairwise spatial correlations as compared with across-piece pairwise spatial correlations, averaged over all emotional events, included the bilateral temporal lobe, the primary and secondary auditory cortex (superior temporal gyrus), and the right anterior temporal lobe. Systematic changes were also shown in the right precentral gyrus and sulcus (Fig. 4*b*). The results were largely the same when using only data from the second half of each event, suggesting the increased similarity in brain patterns in subjects who heard the piece in the same condition is not due to similar fMRI signal spilling over from the event. Taken together, the results suggest that brain representation of emotions in the auditory cortex is sensitive to the affective context in which the stimulus is encountered.

#### Emotional context alters the timing of emotional transitions in the auditory and frontal cortices

**Figure 5.**
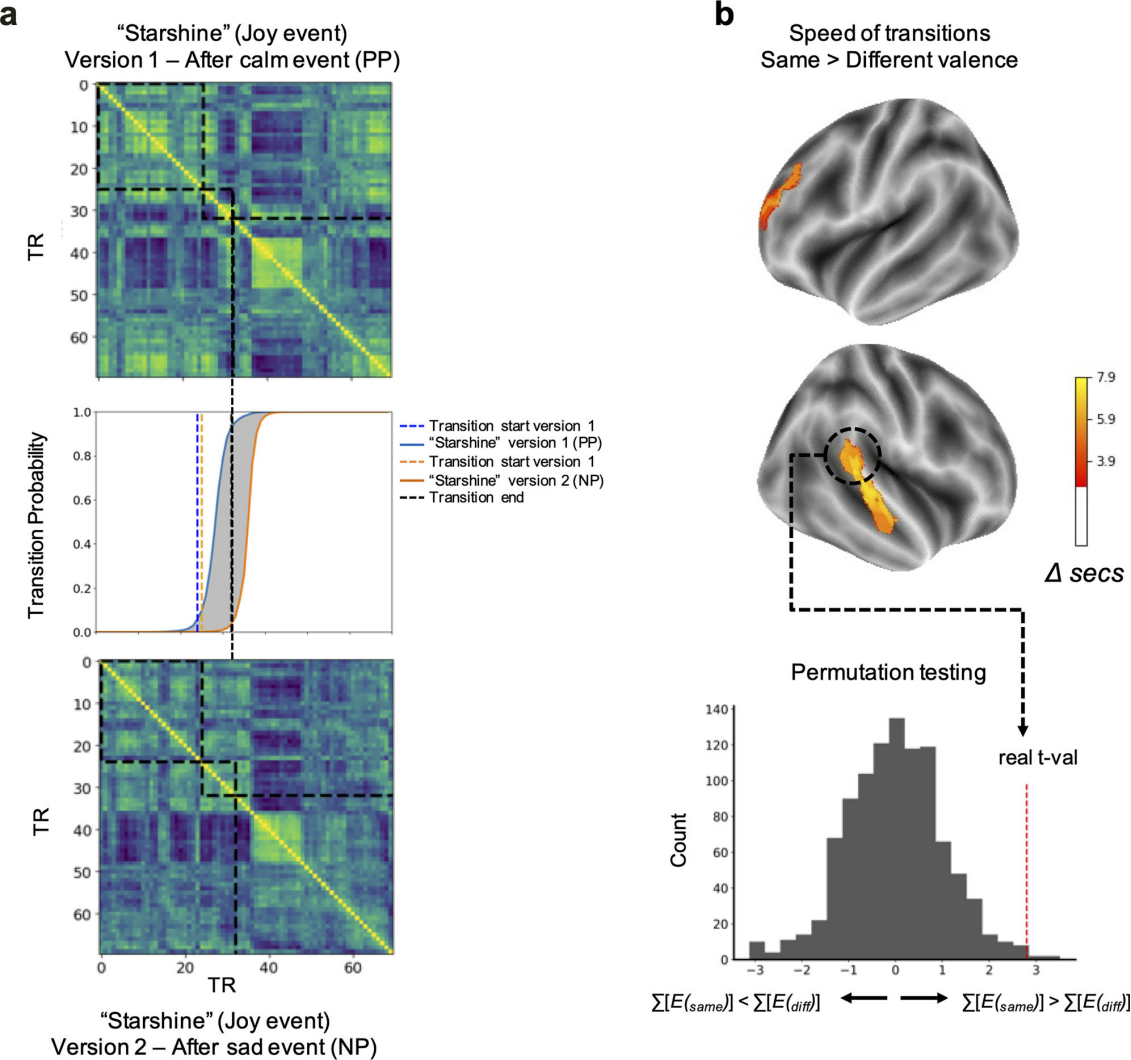
Context-based changes in temporal brain patterns. ***a***, Timepoint-by-timepoint correlations and corresponding HMM-defined transition probability graph for a particular event in participants who heard it in different conditions, showing that when the event was preceded by the same valence (calm event), the transition occurred sooner than when it was preceded by a contrasting valence (sad event). ***b***, Brain regions in which HMM-defined transitions were significantly earlier when the event was preceded by the same valence as compared with a different valence. Colors correspond to the difference in seconds between same valence emotion transitions as compared with different valence transitions (same > different greater than) and were thresholded based on one-sample *t* tests and cluster size at *p* < 0.05.

Across all events, HMM-defined brain-state transitions corresponding to a musical valence shift (negative to positive emotion or positive to negative emotion) occurred later than brain-state transitions corresponding to same valence music transitions (positive to positive or negative to negative, Fig. 5*a*) in surface vertices corresponding to the right auditory cortex and superior temporal gyrus as well as the left superior frontal gyrus (Fig. 5*b*). On average, across all significant brain regions, transitions in which the valence changed occurred 6.26 s later than transitions in which the valence stayed the same.

## Discussion

The aim of this study was to determine how the brain processes the dynamic and contextually dependent nature of emotions. By comparing within-emotional event versus across-emotional event temporal correlations in BOLD signal recorded while participants passively listened to novel, emotionally evocative music, we found that brain-state transitions in voxels in the temporal lobe aligned with composer-defined emotion transitions. Using a probabilistic model, we then determined that emotion dynamics not only contribute to brain-state patterns in these regions but are one of the primary drivers of brain responses to music.

Previous research has highlighted the role of the temporoparietal axis, including the angular gyrus and TPJ, in representing the temporal structure of music (Farbood et al., 2015; Williams et al., 2022) and narratives (Lerner et al., 2011) with short- to long-term information being represented hierarchically along the axis. However, it is unclear from these studies what the nature of the temporal information that is being tracked is. It has been proposed that the superior temporal sulcus in particular, which runs along he temporal lobe, is an important hub for integrating temporal and socioemotional information (Schirmer et al., 2016). Our results provide evidence for this theory and go further, making the case that emotional structure is one of the main organizing principles by which the temporoparietal cortex parses longer temporal experience.

The integral role of the temporoparietal cortex in processing affective experience over time was recently shown in an fMRI study with full-length films (Lettieri et al., 2022). Participants self-reported the emotions that they experienced in real-time while watching two commercially available films, which were subsequently correlated with neural activity collected from a separate group of participants using fMRI. The authors found that functional connectivity (measured with a time-varying intersubject functional correlation analysis) within the temporoparietal cortex, as well as between these regions and the rest of the brain, was correlated with the valence and intensity of emotional responses to the movies. Moreover, the connectivity between different parts of the TPJ and prefrontal regions was associated with emotional responses at different timescales, suggesting that the temporoparietal lobe may represent emotions “chronotopically” (Lettieri et al., 2022). Our results propose a similar role of the temporoparietal axis, yet expand upon the previous findings in several key ways. First, by using musical stimuli written for our study, we can evoke emotional states that are not confounded with semantic properties (such as plot elements) present in films. Second, our results show that there are temporal boundaries between emotion representations in this region, reflecting discrete transitions between affective states rather than simply continuous fluctuations of intensity or valence. Uncovering brain regions sensitive to emotional transitions in particular is an important first step for developing a neural understanding of mood disorders characterized by affective rigidity (Hill et al., 2019; Lydon-Staley et al., 2019; Bylsma, 2021).

With regard to the auditory cortex in particular, prior investigations have shown that this region within the temporoparietal cortex plays an active role in emotion processing and is not solely involved in processing perceptual aspects of sound as once previously thought (Koelsch, 2020). Emotional labels associated with both musical and vocal sounds could be reliably decoded from voxels with the primary and secondary auditory cortex, proposing that these regions represent the emotional content of sounds, independent of their specific acoustic properties (Sachs et al., 2018). Recently, it has been proposed that the auditory cortex may be sensitive to the temporal structure of sounds and music. A fMRI/MEG study showed that the primary auditory cortex is activated earlier in time to nonemotional, musical sounds, whereas the secondary auditory cortex responds more slowly, suggesting chronotopic organization (Benner et al., 2023). Furthermore, fMRI results revealed a shift in auditory processing from posterior to anterior areas as a piece of music transitioned from one music phrase to the next (Burunat et al., 2024). However, no study to date has tried to link the emotional and temporal aspects of the auditory cortex functioning. Here, we show that brain states, and their transitions, within the auditory cortex mirrored the moments when our emotional responses to music changed, suggesting the auditory cortex merges both temporal and affective aspects of our responses to sounds. Furthermore, when acoustic features extracted from the music were regressed out of brain signal, the auditory cortex, and superior temporal gyrus in particular, still reflected the subjective emotional changes, suggesting that acoustic elements of the stimuli were not the sole driver of the time-varying patterns associated with emotional transitions.

By creating two versions of each piece of music that altered the order in which the emotional events were presented, we were additionally able to show that neural response patterns in the auditory cortex, and parts of the temporoparietal cortex, were systematically modulated by the emotional context in which the stimulus was presented to a listener. Behaviorally, retrospective ratings of how a particular musical event made participants feel were more correlated in participants who heard that event within the same condition (i.e., with the same emotional event preceding it) versus in a different condition (i.e., with a different emotional event preceding it). In the brain, spatial correlations in the temporal lobe, including the auditory cortex, were significantly more similar in pairs of participants who heard an emotional event with the same prior context as compared with pairs of participants who heard that event with a different prior context. The results were largely the same when we limited our analysis to only data from the second half of the musical event as well. Taken together, the findings argue that emotion representations in the temporal lobe are sensitive to the previously established emotional context in which music is heard, expanding its previously understood role in emotion and auditory processing to include higher-level, longer-timescale information. In future research, we hope to expand upon this work to explore how far in the past our emotions can influence our current state.

Hidden Markov modeling additionally allowed us to assess “temporal” shifts in brain-state dynamics as a result of emotional context (Lee et al., 2021; Cohen et al., 2022). Previous studies have used such techniques to show that repeated viewing of a movie (Lee et al., 2021) or factors related to aging (Cohen et al., 2022) can temporally shift activity patterns in a way that reflects changes to our subjective experiences. Here, we provide evidence that changes to the “emotional context” in which a piece of music is heard can alter the associated brain dynamics. Brain-state transitions in the right temporal lobe, including the auditory cortex and superior temporal gyrus, occurred earlier in time when the valence of the preceding event was the same as the current. This was also reflected behaviorally, where the time it takes for the same piece of music to evoke an emotion depends on what type of musical emotion came right before it. Put another way, we adjust our emotional response to changes in an external stimulus faster when the upcoming emotion is of a similar valence. This lingering effect of previous emotional states could help explain why changes in emotional valence of music during encoding have been shown to enhance memories for the order and structure of distinct events (McClay et al., 2023).

Our results have broad implications for our understanding of the sociotemporal functioning of the brain and for the field of mental health. Temporal aspects of our everyday emotional states—e.g., their instability, variability, and sustained intensity—seem to play a role in assessing risk for diagnosing and predicting treatment response in mood disorders (Hill et al., 2019). In the case of major depressive disorder, the rigidity affect in daily life may reflect an inability to respond to changing environmental demands (Gruskin et al., 2019). The results presented here may therefore help inform the development of new diagnostic tools and selective treatments for mood disorders. For example, the temporoparietal junction may prove to be a suitable target for neurofeedback paradigms with the goal of moving out of maladaptive states and generating a more flexible emotion system (Johnston et al., 2010). Additionally, similar music-listening paradigms might be used as cheaper and less invasive therapies for treating depression and other mood disorders (Sachs et al., 2015).

Despite our initial hypothesis, subcortical brain areas did not show time-varying brain patterns that reflected music-evoked emotion dynamics. While changes in these regions appear to be reliably predicted by emotional responses evoked by short videos (Horikawa et al., 2020) and in some studies that used music designed to induce emotions (Singer et al., 2016; Koelsch, 2020), other recent evidence found that not all limbic regions reliably represent music-induced emotions (Putkinen et al., 2020) nor musical reward (Mas-Herrero et al., 2021). Furthermore, the majority of studies averaged signal over the duration of the Piece and compared this average signal to some control condition (scrambled music, sine tones, silence, or music designed to convey another emotion). It is therefore possible that these regions have an overall increase in signal in concordance with an affective response, but do not show stable patterns within an emotional event followed by rapid shifting to a new stable pattern. Further work will be needed to determine whether dynamic emotional experiences evoke different kinds of dynamics in subcortical areas, such as transitory responses at boundaries or ramping activity throughout events (Zacks et al., 2010; Tambini et al., 2017).

This study has several limitations. Given the limited number of each type of emotion transitions (a positive valence emotion to a negative valence emotion or a high arousal emotion to a low arousal emotion) in our stimuli set, it was not possible to test the underlying quality of the event transition that may be driving the brain-state changes. Previous research has suggested that changes in context elicit changes in arousal, which segment our memories into separable events (Clewett et al., 2020b). Others have suggested that emotion dynamics arise as a result of the underlying and fluctuating uncertainty associated with trying to predict future outcomes (Majumdar et al., 2023). It could be that specific “types” of emotion transitions (e.g., negative to positive, more arousing to less arousing, or more uncertain to more certain) are specifically driving the event patterns in different ways. Furthermore, the analyses presented here focus on group-level statistics, though it is possible that emotion dynamics are not stable or consistent “across” participants. Follow-up investigations will attempt to assess individual differences in emotional experiences to music using within-subject analyses and richer self-report measures.

In conclusion, by developing novel musical stimuli and employing data-driven methods for capturing dynamic changes in brain and behavior, we show that spatiotemporal patterns along the temporoparietal axis reflect changing emotional experiences to music. Specifically, we found stable brain patterns within the primary auditory cortex, superior temporal gyrus, and sulcus during an emotional event that rapidly shifted to a new stable pattern when the emotional experience induced by music changes. Tempoparietal regions also showed altered spatial and temporal patterns to the same pieces of music that were heard in different emotional contexts. The findings suggest a role of the temporoparietal axis in integrating changing acoustic input with our changing internal states, highlighting a potential neural mechanism by which our emotions fluctuate in everyday life and treatment targets for when such fluctuations go awry in the case of mental illness.

## Synthesis

Reviewing Editor: Luke Remage-Healey, University of Massachusetts Amherst Decisions are customarily a result of the Reviewing Editor and the peer reviewers coming together and discussing their recommendations until a consensus is reached. When revisions are invited, a fact-based synthesis statement explaining their decision and outlining what is needed to prepare a revision will be listed below. The following reviewer(s) agreed to reveal their identity: NONE. Note: If this manuscript was transferred from JNeurosci and a decision was made to accept the manuscript without peer review, a brief statement to this effect will instead be what is listed below.

The manuscript was revised in response to concerns raised by two expert referees. While the authors have addressed the majority of the concerns and critiques, one major issue persists, as indicated by both, and also in earlier reviews: "..the paper does not make a clear case for the results' broad significance." The authors are encouraged to revisit the significance of the work, beyond the novelty of the design/stimuli and cast the findings in a more integrated context for the field.
