# Erratum: Sachs et al., “Emotions in the Brain Are Dynamic and Contextually Dependent: Using Music to Measure Affective Transitions”

**DOI:** 10.1523/ENEURO.0454-25.2025

**Published:** 2026-01-02

In the article, “Emotions in the Brain Are Dynamic and Contextually Dependent: Using Music to Measure Affective Transitions” by Matthew E. Sachs, Mariusz S. Kozak, Kevin N. Ochsner, and Christopher Baldassano published online on June 30, 2025, URLs were omitted from the *Materials and Methods* due to a publisher error. The “Code and data accessibility” subsection should read:

All fMRI images in BIDS format are made available on OpenNeuro at https://openneuro.org/datasets/ds006583/. The code/software described in the paper is freely available online at https://github.com/sachsm53. Audio files of the stimuli are made available on OSF (https://osf.io/a57wu/).

The journal apologizes for this oversight, and the online version has been corrected.

